# Chemotherapy combined with regorafenib and immune checkpoint inhibitors as a first-line treatment for patients with advanced biliary tract cancer: a single arm phase II trial

**DOI:** 10.3389/fimmu.2024.1449211

**Published:** 2024-09-18

**Authors:** Jianwei Liu, Shilei Bai, Yanfu Sun, Lei Hu, Ruiliang Ge, Feng Xue

**Affiliations:** ^1^ Department of Hepatic Surgery II, Third Affiliated Hospital of Naval Medical University (Eastern Hepatobiliary Surgery Hospital), Shanghai, China; ^2^ Department of Hepatic Surgery I, Third Affiliated Hospital of Naval Medical University (Eastern Hepatobiliary Surgery Hospital), Shanghai, China; ^3^ Department of Biliary Tract IV, Third Affiliated Hospital of Naval Medical University (Eastern Hepatobiliary Surgery Hospital), Shanghai, China

**Keywords:** advanced biliary tract cancer, first-line therapy, targeted therapy, immunotherapy, chemotherapy, prognosis

## Abstract

**Objective:**

This study aimed to investigate the efficacy, long-term prognosis and safety of combining chemotherapy with regorafenib and immune checkpoint inhibitors as first-line treatment for patients with advanced biliary tract carcinoma (BTC).

**Methods:**

In this single arm phase II trial, twenty-nine patients with advanced BTC were included, all of whom received gemcitabine-based chemotherapy combined with regorafenib and immune checkpoint inhibitors as the first-line treatment. And the study analyzed anti-tumor efficacy, long-term prognosis, and adverse reactions.

**Results:**

Among the patients, 0 patient achieved complete response, 18 patients (62.1%) achieved partial response, 8 patients (27.6%) had stable disease, and 3 patients (10.3%) experienced progressive disease. The corresponding objective response rate (ORR) was 18/29 (62.1%), and the disease control rate (DCR) was 26/29 (89.7%). The median overall survival (OS) was 16.9 months (95% confidence interval [CI]: 12.0 -21.8) and the median progress free survival (PFS) was 10.2 months (95% CI: 7.8- 12.6). The 1-year OS and PFS were 65% (95% CI: 0.479-0.864) and 41% (95% CI: 0.234-0.656), respectively. The incidence of adverse reactions was 27/29 (93.1%), and the incidence of grade III/IV adverse reactions was 5/29 (17.2%).

**Conclusion:**

The combination of chemotherapy, regorafenib, and immune checkpoint inhibitors as a first-line treatment for patients with advanced BTC may has good anti-tumor efficacy without causing serious adverse reactions, and can significantly improve the long-term prognosis.

## Introduction

Malignant biliary tumors, including gallbladder cancer and cholangiocarcinoma, constitute approximately 3% of all digestive system tumors. Cholangiocarcinoma can be further categorized into intrahepatic cholangiocarcinoma and extrahepatic cholangiocarcinoma (1). At diagnosis, most patients with biliary tract carcinoma (BTC) are in advanced stage, rendering radical surgery unfeasible (2). The prognosis for advanced BTC is extremely poor, with a 5-year overall survival (OS) rate <5% due to the tumor’s aggressive nature. Gemcitabine-based chemotherapy remains the standard first-line treatment and the most common treatment for advanced BTC (3). Studies have reported that the median OS is only 11.7 months for patients with advanced BTC receiving chemotherapy alone (3). Emerging targeted therapies and immunotherapies offer potential improvements in prognosis for advanced BTC patients. The combination of immune checkpoint inhibitors with chemotherapy has shown some promise, with a median OS of 12.8 months, without significantly increasing adverse reactions (4). Additionally, gemcitabine combined with cisplatin and doxorubicin has demonstrated could significantly improve the ORR and PFS in advanced BTC patients (5). Regarding the study of targeted therapy in BTC, one study has shown that pemigatinib can improve the ORR, prolong the sustained remission time, and ultimately improve the long-term prognosis, with a median OS reaching 17.5 months (6). However, the applications of these targeted treatments is often limited to specific patient populations.

Previous studies reported that regorafenib targets multiple tumor pathways involved in BTC progression, including epidermal growth factor receptor (EGFR), rat sarcoma (RAS), rapidly accelerated fibrosarcoma (RAF), vascular endothelial growth factor receptor (VEGFR), fibroblast growth factor receptor (FGFR), and platelet-derived growth factor receptor (PDGFR) signaling pathways (7–10). Previous studies have reported that regorafenib has good efficacy in patients with advanced/metastatic BTC who have failed first-line chemotherapy, with the 12-month survival rate reach 32% (11). Meanwhile, the National Comprehensive Cancer Network guidelines recommend regorafenib as a second-line treatment for BTC (12). However, there is a notable lack of research investigating the first-line treatment of chemotherapy in combination with regorafenib and immune checkpoint inhibitors in patients with advanced BTC. This study aimed to address this gap by exploring the anti-tumor efficacy, long-term prognosis, and adverse reactions associated with gemcitabine-based chemotherapy (gemcitabine and cisplatin) combined with regrafenib and immune checkpoint inhibitors as a first-line treatment for advanced BTC.

## Patients and methods

### Patients

From July 2021 to October 2023, 45 patients with advanced BTC were initially selected in this single arm phase II trial, all of whom received gemcitabine and cisplatin chemotherapy combined with regrafenib and immune checkpoint inhibitor. After the screening of inclusion and exclusion criteria, 29 patients were finally included in this study.

The inclusion criteria of this study were as follows: (1) An aged of 18-80 years; (2) Histologically/cytologically confirmed advanced BTC (intrahepatic, hilar, extrahepatic cholangiocarcinoma, and gallbladder cancer); (3) No prior systemic therapy; (4) No history of other malignancies; (5) Eastern Cooperative Oncology Group (ECOG) score 0 - 1, with a life expectancy > 3 months; (6) Child‒Pugh grade A or B7; (7) According to Response Evaluation Criteria In Solid Tumours (RECIST) 1.1 criteria, there is at least one measurable lesion; (8) Total bilirubin ≤3.0 times the normal upper limit, alanine aminotransferase ≤5.0 times the normal upper limit, international standardized ratio/partial thromboplastin time ≤1.5 times the normal upper limit, serum creatinine level ≤1.5 times the normal upper limit, platelet count ≥75,000/mm^3^, hemoglobin ≥9 g/dL, and absolute neutrophil count ≥1500/mm^3^; and (9) Signed informed consent.

The exclusion criteria were follows: (1) Previous treatment with other anti-tumor therapy; (2) Concurrent other malignant tumors; (3) Incomplete clinical data; (4) Use of targeted treatment other than regorafenib during or prior to the study; (5) Discontinuation of regrafenib for >1 week without justification; (6) Concomitant hematologic disorders or other contraindications for chemotherapy; (7) Use of traditional Chinese medicine treatment during the treatment; (8) Unstable/new angina pectoris or myocardial infarction; (9) Recent bleeding or coagulation disorders; (10) Uncontrolled hypertension (systolic blood pressure ≥140 mmHg or diastolic blood pressure ≥90 mmHg).

All patients provided written informed consent prior to participating in this study, which was approved by the Ethics Committee of the Eastern Hepatobiliary Surgery Hospital (EHBKY2021-H018-P015).

### Treatment procedures

All patients in this study accepted systematic treatment. The systematic treatment included gemcitabine and cisplatin chemotherapy, regorafenib and immune checkpoint inhibitors. The chemotherapy protocol consisted of gemcitabine 1000 mg/m² and cisplatin 25 mg/m² administered intravenously on days 1 and 8 of 3-week cycles. Targeted treatment protocol was follow: regorafenib, 80 mg/day, orally, days 1-21 of 4 weeks cycles. Immunotherapy protocol was follow: programmed cell death protein 1/programmed cell death ligand 1 (PD-1/PD-L1) included Durvalumab (1500 mg) or Pembrolizumab (200 mg) or Tislelizumab (200 mg), which were administered on day 1 of each cycle before chemotherapy. Chemotherapy was administered intravenously on a 21-day cycle for up to eight cycles. Then subsequent maintenance therapy includes immunotherapy and targeted therapy. Immunotherapy included Durvalumab (1500 mg) or Pembrolizumab (200 mg) or Tislelizumab (200 mg) administered intravenously once every 3 weeks combined with regorafenib which was administered orally (80 mg/day) days 1-21 of 4 weeks cycles until clinical or imaging disease progression or until unacceptable toxicity, or any other discontinuation criteria were met. Blood routine, liver, and kidney function tests were conducted before each treatment cycle.

### Follow-up, safety and efficacy assessment

Systemic therapy continued until patients occurred serious adverse reactions, tumor progression, or death. All patients were followed up regularly, and adverse reactions events were recorded according to the National Cancer Institute’s Common Terminology Criteria for Adverse Events, version 4.0 (13). Efficacy assessments included liver and renal function tests, blood routine tests, serum tumor markers [alpha-fetoprotein (AFP), carcinoembryonic antigen (CEA), carbohydrate antigen 199 (CA-199)], and liver magnetic resonance imaging (MRI) every 2 months. Anti-tumor efficacy was evaluated using the RECIST 1.1 criteria (14). The best therapeutic effect was classified as follows: Complete response (CR): complete disappearance of all target lesions, with no new lesions, maintained for > 4 weeks. Partial response (PR): tumor shrinkage >30%, maintained for >4 weeks. Stable disease (SD): tumor shrinkage of no more than 30% or increase of no more than 20%. Progressive disease (PD): Tumor increase >20% or appearance of new lesions. The ORR was defined as CR+PR, which refers to the probability of tumor shrinkage. The DCR included CR+PR+SD, which refers to the probability of tumor shrinking or maintaining stability. Long-term prognosis was assessed by OS and PFS. OS was defined as the time from the patient’s initial diagnosis to death or last follow-up. PFS was defined as the time from the treatment to tumor progression or death.

### Statistical analysis

Measurement data were described by median (range). Count data were described by frequencies (percentage). The OS and PFS curves were plotted by the Kaplan-Meier method. All data were analyzed by SPSS software (version 26, SPSS INC., Chicago, IL, USA).

## Results

### Patient characteristics

**Figure 1:**
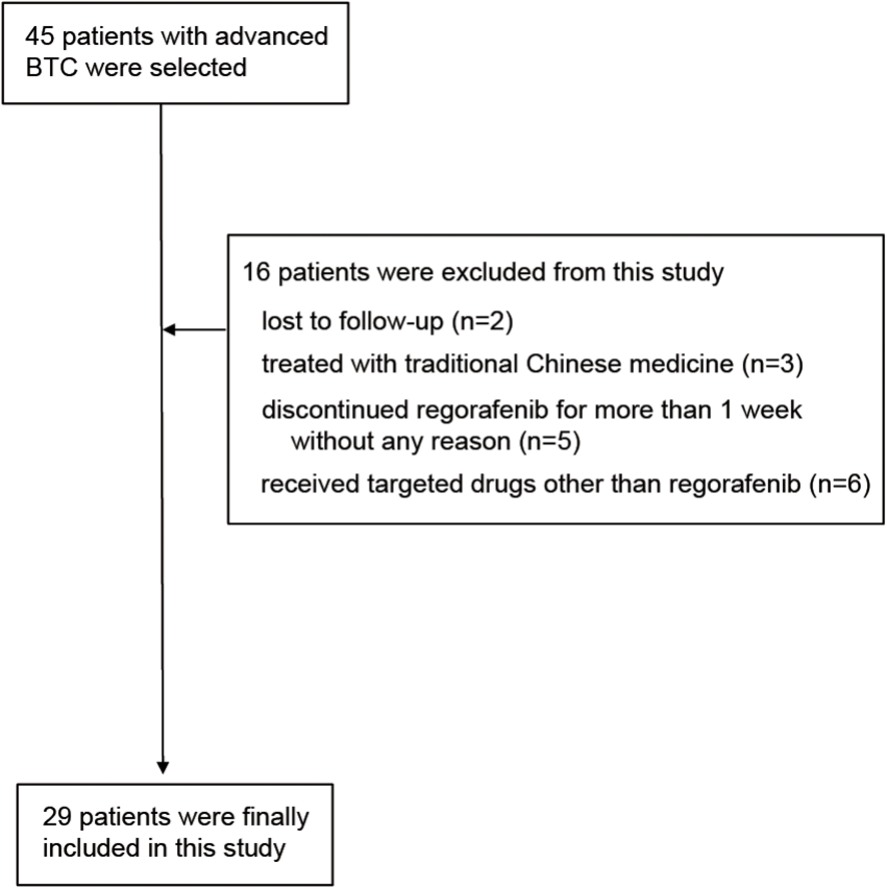
The flow chart of this study.

**Figure 1** shows the flow chart of this study. Initially 45 patients were enrolled in the study. After screening by inclusion exclusion criteria, 29 patients were finally included in this study. The reasons for exclusion were: lost to follow-up (2 patients), treated with traditional Chinese medicine (3 patients), discontinuation of regorafenib for >1 week without justification (5 patients), and receipt of targeted drugs other than regorafenib (6 patients).

**Table 1** shows the basic information of the 29 patients included in this study. The mean age was 58 (interquartile range: 50-66.5) years. There were 19 men and 10 females. Among the 29 patients, there were gallbladder cancer (6 cases), intrahepatic cholangiocarcinoma (14 cases), and extrahepatic cholangiocarcinoma (9 cases). The ECOG scores were 0 in 16 patients and 1 in 13 patients, respectively. The total bilirubin (TBIL) level was 16.4 (11.9-39.1) μmol/L, albumin (ALB) was 39.1 (37.4-44.7) g/L, alanine transaminase (ALT) was 38.0 (11.0-146.0) U/L, aspartate aminotransferase (AST) was 33.0 (18.0-102.0) U/L, platelet (PLT) was 165 (116.5-210.5)/L, prothrombin time (PT) was 11.8 (11.1-12.5) s, alpha fetoprotein (AFP) was 4.3 (2.6-15.2) μg/L, carcinoembryonic antigen (CEA) was 2.5 (1.4-3.3) ng/ml, carbohydrate antigen199 (CA199) was 285.4 (73.4-868.5) U/ml, Protein Induced by Vitamin K Absence or Antagonist-II (PIVKA II) was 34.0 (12.0-900.0) μg/L. Among 29 patients, 5 were positive for hepatitis B surface antigen, 6 had cirrhosis, 19 had vascular invasion, 19 had lymph node metastasis, and 23 had extrahepatic metastasis. The patients with high, stable, and missing microsatellite instability (MSI) status were 1 (3.4%), 17 (62.1%), and 11 (34.5%), respectively. And the expression level of PD-L1 was as follows: 15 patients (51.7%) with TAP ≥1%, 10 patients (34.5%) with TAP <1%, and 4 patients (13.8%) with PD-L1 missing.

**Table 1:** Baseline characteristics of patients.

Variables	Number (%)/median (IQR)	Variables	Number (%)/median (IQR)
Age, years	58.0 (32.0-77.0)	Tumor type	
Sex		Gallbladder cancer	6 (20.7)
Male	19 (65.5)	ICC	14 (48.3)
Female	10 (34.5)	ECC	9 (31.0)
ECOG score		Tumor diameter, cm	4.6 (1.5-13.8)
0	16 (55.2)	Tumor number	
1	13 (44.8)	Single	5 (17.2)
Child Pugh		Multiple	24 (82.8)
A	26 (89.7)	Vascular invasion	
B	3 (10.3)	No	10 (34.5)
TBIL, μmol/L	16.4 (6.7-60.0)	Yes	19 (65.5)
ALT, U/L	38.0 (11.0-146.0)	Lymph node metastasis	
AST, U/L	33.0 (18.0-102.0)	No	10 (34.5)
ALB, g/L	39.1 (31.2-46.1)	Yes	19 (65.5)
PLT, 10^9^ /L	165.0 (73.0-279.0)	Extrahepatic metastasis	
PT	11.8 (11.0-14.0)	No	6 (20.7)
HBsAg		Yes	23 (79.3)
Negatives	24 (82.8)	MSI status	
Positive	5 (17.2)	High	1 (3.4)
Cirrhosis		Stable	17 (62.1)
No	23 (79.3)	Missing*	11 (34.5)
Yes	6 (20.7)	PD-L1 expression	
AFP, μg/L	4.3 (1.2-960.0)	TAP ≥1%	15 (51.7)
CEA, ng/ml	2.5 (0.9-26.2)	TAP <1%	10 (34.5)
CA 199, U/mL	285.4 (0.2-1000.0)	Missing	4 (13.8)
PIVKA II, μg/L	34.0 (12.0-900.0)		

IQR, interquartile range; ECOG, Eastern Cooperative Oncology Group; TBIL, total bilirubin; ALT, alanine transaminase; AST, aspartate aminotransferase; ALB, albumin; PLT, platelet; PT, prothrombin time; HBsAg, hepatitis B surface antigen; AFP, alpha fetoprotein; CEA, carcinoembryonic antigen; CA 199, carbohydrate antigen199; PIVKA II, Protein Induced by Vitamin K Absence or Antagonist-II; ICC, intrahepatic cholangiocarcinoma; ECC, extrahepatic cholangiocarcinoma; MSI, microsatellite instability; PD-L1 programmed cell death ligand 1; TAP tumor area positivity (proportion of tumor and/or immune cells with PD-L1 staining at any intensity).

*MSI status missing includes MSI-unknown and not tested.

### The effectiveness of systemic treatment

**Figure 2:**
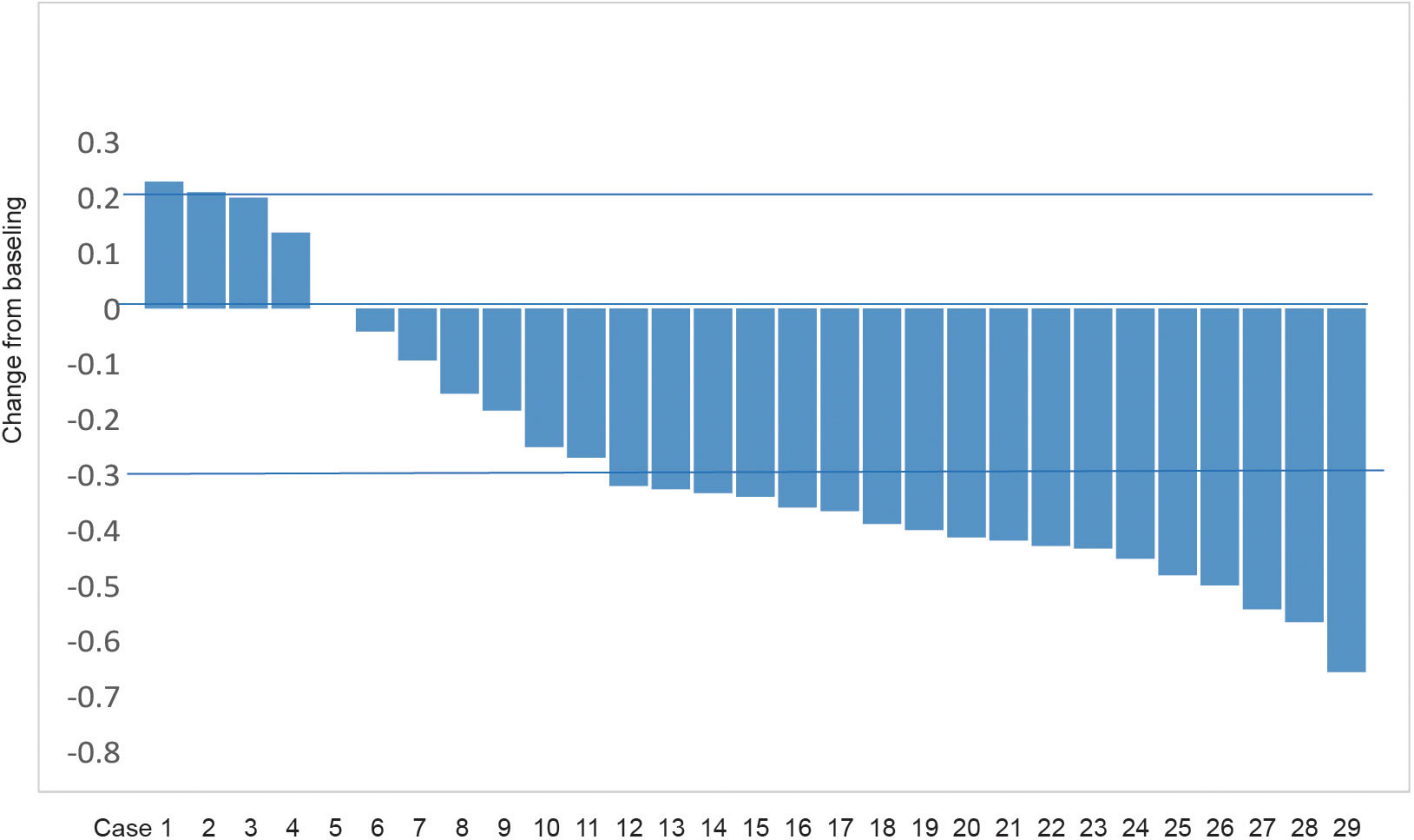
Waterfall plot showing maximum tumor change of target lesions.

Of the 29 patients included in this study, the number of patients with CR, PR, SD, and PD was 0, 18 (62.1%), 8 (27.6%), and 3 (10.3%) according to RECIST 1.1 criteria. The corresponding ORR was 62.1% and DCR was 89.7% (**Table 2**). **Figure 2** presents the waterfall plot of the changes in tumor diameter before and after systemic therapy of the 29 patients.

**Table 2:** The anti-tumor efficacy and tumor response parameters of these patients.

Variables	Number (%)	Variables	Parameters
CR	0	Median duration of response (95% CI), months	10.5 (6.7-14.3)
PR	18 (62.1)		
SD	8 (27.6)	Median time-to-response (95% CI), months	1.8 (1.3-2.2)
PD	3 (10.3)	Patients with continued response (%)	
ORR	18 (62.1)	≥3	96.6
DCR	26 (89.7)	≥6	82.8
		≥9	65.5
		≥12	44.8

CR, complete response; PR, partial response; SD, stable disease, PD, progressive disease; ORR, objective response rate; DCR, disease control rate; CI, confidence intervals.

### The prognosis of systemic therapy

**Figure 3:**
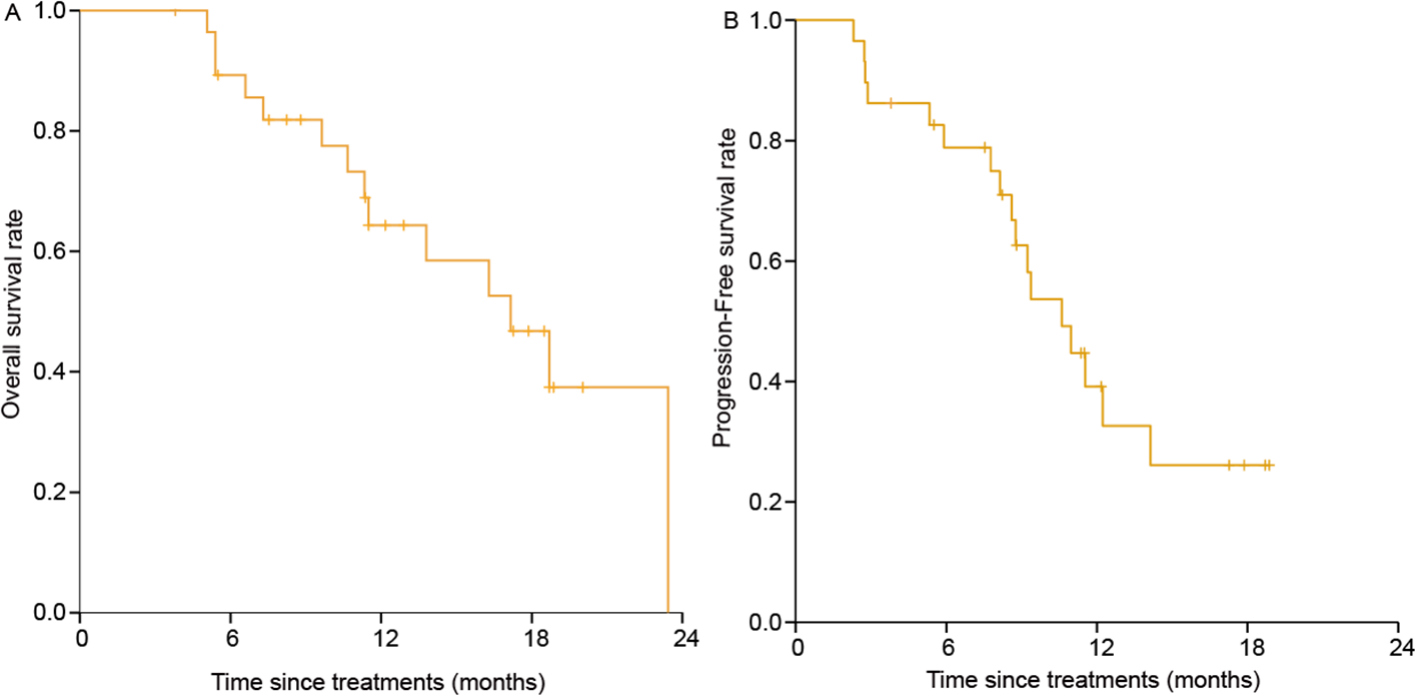
Kaplan-Meier Curves of overall survival and Progression-free survival rate.

The median follow-up duration was 17.8 months (95% CI: 9.9-25.8). At the end of follow-up period, 17 patients had tumor progression, 14 had died, and 15 were still alive. The median duration of response was 10.5 months (95% CI: 6.7-14.3) and the median time-to-response was 1.8 months (95% CI: 1.3-2.2). The percentage of patients with continued response for ≥3, ≥6, ≥9 and ≥12 months were 96.6%, 82.8%, 65.5% and 44.8% (**Table 2**). The median OS and PFS was 16.9 months (95% CI: 12.0-21.8) and 10.2 months (95% CI: 7.8-12.6). The 1-year OS and PFS were 65% (95% CI: 0.479-0.864) and 41% (95% CI: 0.234-0.656) (**Figure 3**). Subgroup analysis was did according to intrahepatic cholangiocarcinoma, extrahepatic cholangiocarcinoma and gallbladder cancer, and the Kaplan–Meier curves of different tumors are shown in **Supplementary Figure 1**.

### The safety of systemic therapy

**Table 3** shows the adverse reactions observed in 29 patients. A total of 27 patients experienced 60 adverse reactions, with 11 patients having 3 adverse reaction, 11 having 2 adverse reaction, and 5 having 1 adverse reaction. The overall incidence of adverse reaction was 27/29 (93.1%), and the incidence of grade III/IV adverse reaction was 5/29 (17.2%). The most common adverse reaction included gastrointestinal reaction (19/29), hand-foot skin reaction (9/29), bone marrow suppression (7/29), and hepatic impairment (7/29).

**Table 3:** The summary of adverse reactions after receiving systemic therapy.

Adverse reactions	Incidence, n=29 (%)	
	All grades (%)	≥III grades (%)
**Total patients**	27 (93.1) ^‡^	5 (17.2) ^&^
**Total events**	60	7
Gastrointestinal reaction	19 (65.5)	0
Hand-foot skin reaction	9 (31.0)	3 (10.3)
Bone marrow suppression	7 (24.1)	1 (3.4)
Fatigue	4 (13.8)	0
Liver function damage	7 (24.1)	2 (6.8)
Hypertension	6 (20.7)	1 (3.4)
Diarrhea	3 (10.3)	0
Weight loss	3 (10.3)	0
Other	2 (6.9)	0

^‡^: one, two and three types of adverse reaction occurred in 5, 11 and 11 patients, respectively.

^&^: one and two types of adverse reaction occurred in 3 and 2 patients, respectively.

## Discussion

The prognosis for patients with advanced BTC remains poor, and how to improve the prognosis of these patients was the difficulty and focus of clinical research (15). Current treatment protocols for advanced BTC include gemcitabine and cisplatin chemotherapy, FOLFOX chemotherapy, and so on (16). However, these treatments still results in unsatisfactory prognoses (3, 17). The TOPAZ-1 and KEYNOTE-966 trials has changed the situation where advanced BTC are only treated with chemotherapy (18, 19). At the same time, it also ushered in a new era of immunotherapy for patients with advanced BTC. However, the study of targeted therapy for these patients is still lacking. Previous research demonstrated that GEMOX chemotherapy combined with donafenib and immunotherapy showed better anti-tumor efficacy in advanced BTC (20). Similarly, other studies reported that lenvatinib combined with PD-1 achieved ORR of 9.7-30.4% and DCR of 67.7-85.7% (21, 22). The prognosis of these studies are significantly better than those of the current standard first-line treatments (3).

Our study systematically analyzed the efficacy and safety of gemcitabine and cisplatin chemotherapy combined with immunotherapy and targeted therapy for advanced BTC. Compared to the TOPAZ-1 and KEYNOTE-966 trials, our study added a targeted drug. Regorafenib is a second-line targeted drug for advanced BTC in NCCN guidelines, and is also a multi kinase multi-target drug. In the TOPAZ-1 and KEYNOTE-966 trials, there were two patients (0.6%) and seven patients (1%) in the chemotherapy group assessed as CR (18, 19). There were indeed some patients assessed as CR in both trials. However, it can be seen that a relatively large number of patients were included in these two trials. In clinical, we have indeed observed that a small number of patients are very sensitive to certain treatments such as chemotherapy, but the specific reasons are still being explored. In this study, no patients achieved CR, which may be due to the relatively small number of patients included in this study. Although no patients were assessed as CR, the combination yielded an ORR of 62.1% and a DCR of 89.7%, with only 3 patients were evaluated as PD. These results proved that this combination treatment offers a superior anti-tumor efficacy compared with previous studies (3, 20–22). In addition, in terms of long-term prognosis, our study showed that the median OS and PFS were 16.9 months and 10.2 months, respectively, which was significantly higher than previously reported data. This may be attributed to the better anti-tumor effect of the chemotherapy combined with regorafenib and immunotherapy. We analyzed the possible reasons for the better anti-tumor efficacy and long-term prognosis of this combination treatment. Firstly, previous studies reported that regrafenib targets multiple tumor pathways involved in biliary tumorigenesis, including EGFR, RAS, RAF, VEGFR, FGFR, and PDGFR signaling pathways (7–10). By targeting these pathways, regorafenib may induce rapid tumor death and antigen release, which could enhance the immune response and improve the efficacy of immunotherapy (23). Additionally, chemotherapy may reduce the immunosuppressive effects of the tumor microenvironment, facilitate antigen cross-presentation, and increase the infiltration of immune cells (24). Thus, targeted drugs combined with immunotherapy and chemotherapy may provide synergistic benefits and could theoretically improve the response to anti-tumor therapy. It may be due to the good anti-tumor effect. Our study reported that the median OS of 16.9 months and the median PFS of 10.2 months, which surpasses the 12.8 months reported for combined chemotherapy and immunotherapy in previous studies (4). Besides, there were 15 patients (51.7%) with TAP ≥1% and 10 patients (34.5%) with TAP <1% in our study. The proportion of patients with TAP ≥1% in this study was lower that TOPAZ-1 trial. However, as we know, there is still a lack of evidence regarding the relationship between PDL-1 expressions and the prognosis and response of BTC. In this study, we also attempted to analyze the survival differences and anti-tumor efficacy of patients accepting different immune checkpoint inhibitors, but due to the relatively small number of patients, we have only presented the prognosis curves (**Supplementary Figure 2**) and the anti-tumor efficacy and long-term prognosis of different immune therapies (**Supplementary Table 1**).

Regarding safety, our result showed that the overall incidence of adverse reactions was 27/29 (93.1%), and the incidence of grade III/IV adverse reactions was 5/29 (17.2%). The overall incidence of adverse reactions was similar to that reported in previous studies (18, 19). This result shows that the combined treatment will not increase the drug toxicity, and will not lead to serious adverse reactions, which is consistent with the previous reports that chemotherapy combined with target immunization therapy will not significantly increase the drug toxicity of patients (25). However, the incidence of grade III/IV adverse reactions was lower than previous studies in our study. This may be attributed to our intervention measures for adverse reactions. In our study, gastrointestinal reactions were the most common adverse reactions, with 19/29 (65.5%) patients experiencing gastrointestinal reactions, which often caused by chemotherapy (26). Among them, 18/29 (62.6%) patients were assessed as grade II instead of grade III/IV in this study, which may be attributed to our management of adverse reactions. When patients experienced mild or very mild symptoms, we provided corresponding gastrointestinal interventions in advance, including dietary adjustments and medication adjustments (such as acid suppression, antiemetic, bowel movements, digestion promotion, etc.). Therefore, these 18 patients did not experience any serious complications (≥grade III) that would affect their daily life or subsequent anti-tumor therapy. If these patients did not undergo early intervention and treatment, most of them will be hospitalized or have subsequent anti-tumor therapy suspended due to serious adverse reactions. Of course, the majority of patients (55.2%) in this study had an ECOG score 0, which is relatively better than the ECOG status of patients included in the previous two trials (18, 19). Meanwhile, the patients included in this study were relatively young than the patients of other two trials (18, 19). The overall condition of the enrolled patients is better, and their tolerance is relatively better. This is also the reason why there are fewer grade III/IV adverse reactions reported in this study. These patients ultimately did not reduce or even stop medication due to serious adverse reactions, which may also be the reason for the better anti-tumor efficacy and long-term prognosis.

Limitations of this study include the relatively small sample size, the single-center design and the data lack of randomization. However, these patients included in this study were enrolled continuously without any human selection factors, which to some extent avoided selection bias.

## Conclusion

The combination of gemcitabine and cisplatin chemotherapy with regorafenib and immunotherapy as a first-line treatment for patients with advanced BTC may have better anti-tumor efficacy, patients could achieve a better long-term prognosis, and does not significantly increase the adverse reactions. Although our study was a phase II study. From the perspective of data rigor, we cannot claim that our data is superior to previous studies because of the lack of randomization. Our study only provides a systematic treatment protocol that may achieve good therapeutic effects. Further large-scale multicenter phase III clinical studies are still needed to confirm our conclusion.

## Data Availability

The raw data supporting the conclusions of this article will be made available by the authors, without undue reservation.
