# Supplementary figures and images for: Chemotherapy combined with regorafenib and immune checkpoint inhibitors as a first-line treatment for patients with advanced biliary tract cancer: a single arm phase II trial
Source: Front Immunol. 2024 Sep 18;15:1449211. doi: 10.3389/fimmu.2024.1449211 (PMC11445073; doi:10.3389/fimmu.2024.1449211)

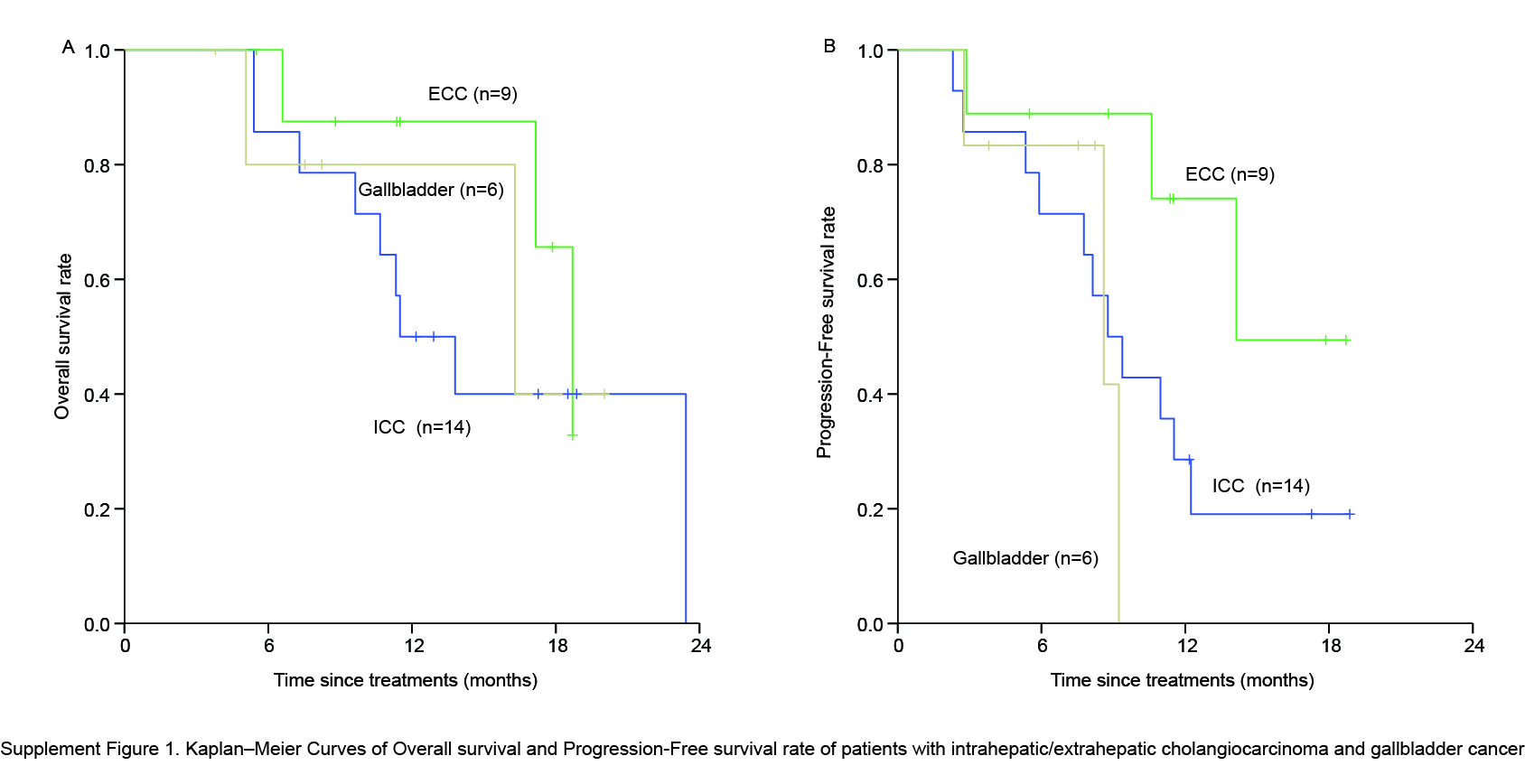

Supplement: Supplementary file 1 [file Image1.tif]

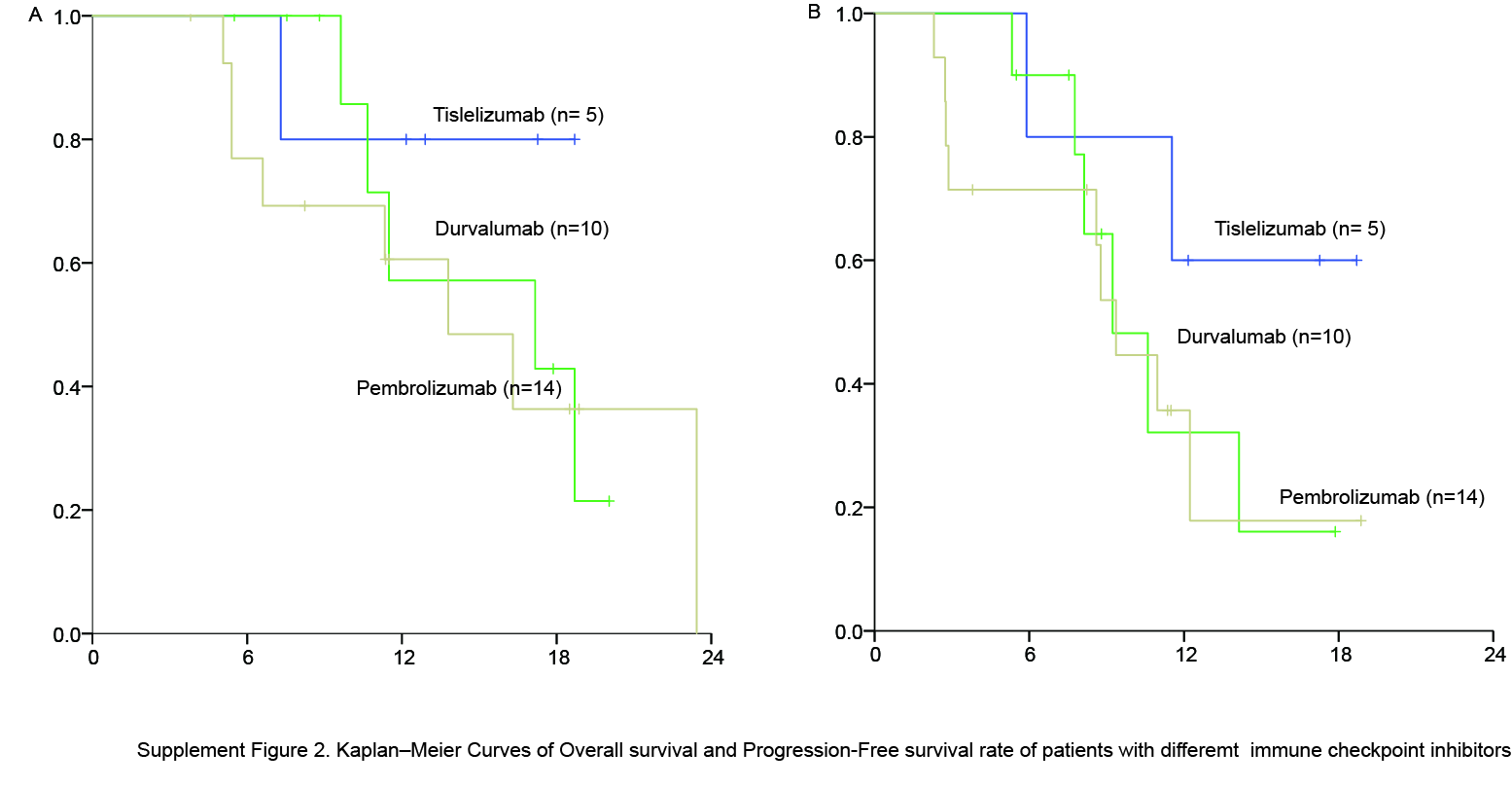

Supplement: Supplementary file 2 [file Image2.tif]
