# Supplementary material for: Chemotherapy combined with regorafenib and immune checkpoint inhibitors as a first-line treatment for patients with advanced biliary tract cancer: a single arm phase II trial
Source: Front Immunol. 2024 Sep 18;15:1449211. doi: 10.3389/fimmu.2024.1449211 (PMC11445073; doi:10.3389/fimmu.2024.1449211)
Supplement: Supplementary file 3 [file Table1.docx]

**Supplement Table 1. The anti-tumor efficacy and long-term prognosis of patients accepting different immune checkpoint inhibitors**

| Variables | Number (%) | Variables | Number (%) |
| --- | --- | --- | --- |
| **Tislelizumab (n=5)** |  | **Pembrolizumab (n=14)** |  |
| CR | 0 | CR | 0 |
| PR | 3 (60.0) | PR | 8 (57.1) |
| SD | 1 (20.0) | SD | 4 (28.6) |
| PD | 1 (20.0) | PD | 2 (14.3) |
| ORR | 3 (60.0) | ORR | 8 (57.1) |
| DCR | 4 (80.0) | DCR | 12 (85.7) |
| mOS | NA | mOS | 16.3 (9.4-23.2) |
| mPFS | NA | mPFS | 9.4 (8.0-13.2) |
| **Durvalumab (n=10)** |  |  |  |
| CR | 0 |  |  |
| PR | 7 (70.0) |  |  |
| SD | 3 (30.0) |  |  |
| PD | 0 (10.3) |  |  |
| ORR | 7 (70.0) |  |  |
| DCR | 10 (100) |  |  |
| mOS | 17.2 (2.6-31.7) |  |  |
| mPFS | 9.2 (6.4-12.1) |  |  |
| **Abbreviations:** CR, complete response; PR, partial response; SD, stable disease, PD, progressive disease; ORR, objective response rate; DCR, disease control rate; mOS, median overall survival; mPFS, median progression-free survival. | | | |
